# Tumor mutational burden quantification from targeted gene panels: major advancements and challenges

**DOI:** 10.1186/s40425-019-0647-4

**Published:** 2019-07-15

**Authors:** Laura Fancello, Sara Gandini, Pier Giuseppe Pelicci, Luca Mazzarella

**Affiliations:** 1 Department of Experimental Oncology, IEO, European Institute of Oncology IRCCS, Via Adamello 16, 20139 Milan, Italy; 2 Department of Oncology and Hemato-Oncology, University of Milan, via Santa Sofia 9, 20142 Milan, Italy; 3 Division of Early Drug Development, IEO, European Institute of Oncology IRCCS, Milan, Italy

**Keywords:** Tumor mutational burden, TMB, Gene panels, Targeted enrichment sequencing, Immunotherapy

## Abstract

Tumor mutational burden (TMB), the total number of somatic coding mutations in a tumor, is emerging as a promising biomarker for immunotherapy response in cancer patients. TMB can be quantitated by a number of NGS-based sequencing technologies. Whole Exome Sequencing (WES) allows comprehensive measurement of TMB and is considered the gold standard. However, to date WES remains confined to research settings, due to high cost of the large genomic space sequenced. In the clinical setting, instead, targeted enrichment panels (gene panels) of various genomic sizes are emerging as the routine technology for TMB assessment. This stimulated the development of various methods for panel-based TMB quantification, and prompted the multiplication of studies assessing whether TMB can be confidently estimated from the smaller genomic space sampled by gene panels. In this review, we inventory the collection of available gene panels tested for this purpose, illustrating their technical specifications and describing their accuracy and clinical value in TMB assessment. Moreover, we highlight how various experimental, platform-related or methodological variables, as well as bioinformatic pipelines, influence panel-based TMB quantification. The lack of harmonization in panel-based TMB quantification, of adequate methods to convert TMB estimates across different panels and of robust predictive cutoffs, currently represents one of the main limitations to adopt TMB as a biomarker in clinical practice. This overview on the heterogeneous landscape of panel-based TMB quantification aims at providing a context to discuss common standards and illustrates the strong need of further validation and consolidation studies for the clinical interpretation of panel-based TMB values.

**Electronic supplementary material:**

The online version of this article (10.1186/s40425-019-0647-4) contains supplementary material, which is available to authorized users.

## Tumor mutational burden: an emerging biomarker for cancer immunotherapy

Immunotherapy with immune checkpoint inhibitors targeting cytotoxic T lymphocyte associated 4 (CTLA-4) or programmed cell death 1 (PD-1) or its ligand (PD-L1) can provide important clinical benefit to patients affected by multiple cancers, most notably lung cancer [1, 2], melanoma [3], renal cancer [4] and urothelial carcinoma [5]. However, only a fraction of patients currently treated by immune checkpoint inhibitors derive benefit from it, while a minority of them suffers from severe side effects. Given the significant cost and non-negligible toxicity of these therapies, the identification of strategies to adequately select those patients most likely to show a favorable response is recognized as an urgent medical need. A few potential biomarkers have been identified up to now, such as PD-L1 gene expression, microsatellite instability (MSI), mismatch repair deficiency (dMMR), POLE or JAK1/2 mutations, immune cell infiltration, IFNγ expression, tumor mutational burden (TMB) or neoantigen burden [6, 7].

**Fig. 1:**
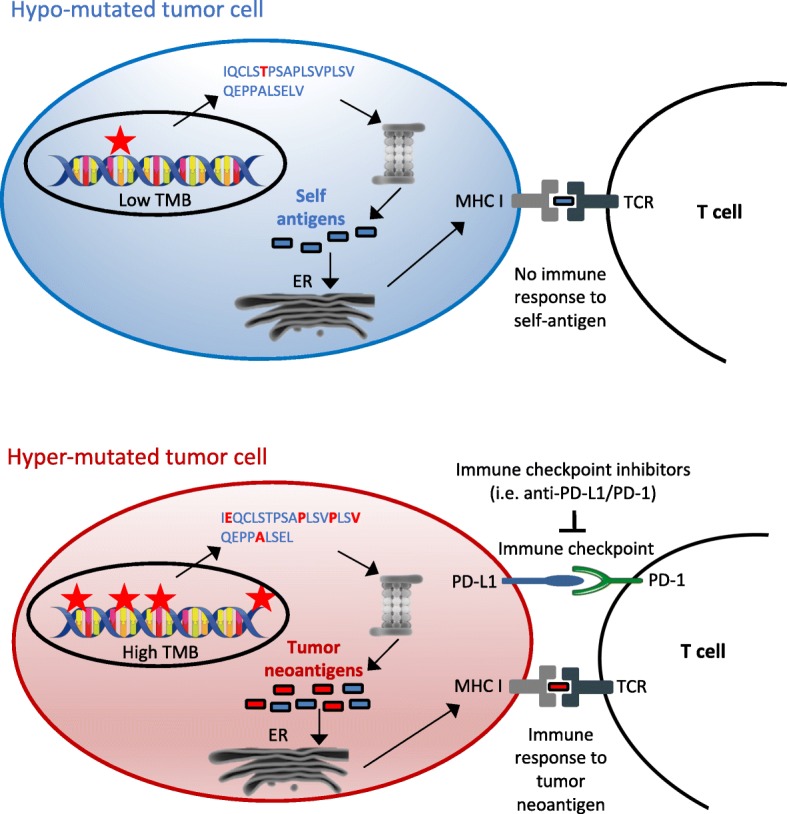
Tumor mutational burden as immunotherapy biomarker. Interaction between tumor mutational burden, neoantigen production and immune checkpoints. Hyper-mutated tumors (bottom) are more likely than hypo-mutated tumors (top) to generate tumor-specific peptides (neoantigens) recognized by the immune system. However, immune surveillance can be restrained by simultaneous high expression of PD-L1, which delivers a suppressive signal to T cells. PD-L1/PD-1 interaction and other immune checkpoints can be inhibited by immune checkpoint inhibitors, restoring immune response

TMB is a measure of the total amount of somatic coding mutations in a tumor and it is currently investigated as a potential biomarker in non-small cell lung carcinoma (NSCLC) [8–10]. Accumulating evidence, however, suggests its potential usefulness also in melanoma [8, 11–14], urothelial cancer [5, 15, 16], mismatch-repair deficient colorectal tumors [17] and other cancer types [18]. Its pattern and distribution is highly variable across different cancer types, with over 1000-fold difference between cancer types with the lowest mutational burden and those with the highest mutational burden, such as those associated with DNA environmental damage, i.e. by exposure to tobacco smoke or UVs [19, 20]. Increased TMB was also observed in tumors with defects in DNA mismatch repair and DNA replication or in tumors characterized by microsatellite instability, as in colorectal cancer [21, 22]. Highly mutated tumors are more likely to produce abundance of tumor-specific mutant epitopes, which may function as neoantigens recognized as non-self by the immune system. Therefore, increased activation of immune cells by treatment with immune checkpoint inhibitors may lead to improved immune-mediated tumor-cell clearance and clinical response in these tumors (Fig. 1). A significant association between neoantigen production and immune-mediated clinical response was indeed observed in several studies [9, 11, 14, 23]. Measurement of this neoantigen production, though, is expensive and time-consuming. Tumor neoantigens can be generated by mutations or by gene fusions, especially out-of-frame fusions. Although some pipelines have recently been developed for the identification of neoantigens derived from gene fusions [24], most research up to now has estimated overall neoantigen load based only on somatic nonsynonymous coding mutations, called by Whole Exome Sequencing (WES). Briefly, somatic nonsynonymous coding mutations are identified by WES and, if RNA sequencing is also available, only mutations occurring in expressed genes are retained. Peptides containing selected mutations are then identified in silico and the efficiency of their presentation to the immune system may be evaluated by mass spectrometry or by algorithms that consider their predicted affinity to the MHC class I complex and patient-specific HLA class I alleles [14, 25]. In comparison with overall neoantigen load, TMB is easier to measure and correlates with it. Although not all mutations can give rise to tumor immunogenic peptides, their number influences the amount of neoantigens potentially produced. High TMB correlates with long-term clinical benefit from immune checkpoint inhibitors in patients with melanoma [14], NSCLC [9, 26–28] and urothelial carcinoma [5, 15, 16, 29]. In addition to that, patients with mismatch repair (MMR) deficient tumors are more responsive to immunotherapy, probably due to their high tumor mutational burden [17]. Therefore, although not always capable to explain the clinical benefit alone, TMB is a good approximation for neoantigen load assessment [14], is technically less challenging and less expensive and may represent a better suited predictive biomarker for immunotherapy response.

TMB may also represent a relevant prognostic biomarker. In BRCA-1/2 mutated ovarian cancers, TMB correlates with improved overall survival [30, 31]. In breast cancer patients, tumors with high TMB and favorable immune-infiltrate (“hot tumors”) are associated with prolonged survival [32]. Consistently, basal cell carcinoma, which is characterized by very high TMB, presents with slow growth rates and rare metastases. Although not definitively demonstrated, we can speculate that this less aggressive phenotype may be due to hypermutation, which would trigger, via increased neoantigen production, a more effective immune response of the host [33].

## Quantification of tumor mutational burden from gene panels: “yes we can”

Initial studies showing a correlation between TMB and enhanced response to immunotherapy were based on WES datasets for TMB quantification [9, 14, 17]. WES allows a direct measurement of TMB, yet it remains unsuitable as routine technology in clinical practice, because expensive, labor-intensive and time-consuming. Therefore, several studies explored the possibility to provide equally accurate and clinically predictive TMB estimates from targeted enrichment sequencing, using various gene panels (Table 1, Additional file 1: Table S1).

**Table 1:** Overview of the main published studies on TMB quantification from gene panels

Reference	Gene panel (version)	Cancer type	Study design	Study ID	ICI	TMB cutoff (mut/Mb)	Method of TMB cutoff determination	TMB predictive value	Clinical outcome	N patients
Rosenberg, 2016 [5]	FM1 (v3)	urothelial carcinoma (metastatic or locally advanced)	trial (single-arm, phase 2)	NCT02108652	PD-(L)1	NA	NA	NA	ORR	315
Balar, 2017 [16]	FM1±	urothelial carcinoma (metastatic)	trial (single-arm, phase 2)	NCT02108652	PD-(L)1	Q3 (> = 16)	distribution	NA	OS	123
Powles, 2018 [15]	FM1±	urothelial carcinoma (metastatic)	trial (randomized, phase 3)	NCT02302807	PD-(L)1	Q2 (9.65)	distribution	NA	OS	931
Kowanetz, 2016 [27]	FM1 (v3)	NSCLC	trial (randomized, phase 2)	NCT01903993	PD-(L)1	Q1, Q2 (9.9), Q3	distribution	NA	PFS, OS, ORR	454
			trial (single-arm, phase 2)	NCT02031458						
			trial (single-arm, phase 2)	NCT01846416						
Gandara, 2018 [61] ^a^	FM1 bTMB assay	NSCLC	trial (randomized, phase 2)	NCT01903993	PD-(L)1	> = 14	positive and negative percentage agreement with the orthogonally validated FM1	NA	PFS, OS	259
			trial (randomized, phase 3)	NCT02008227						
Hellmann, 2018 [50]	FM1 CDx	NSCLC	trial (randomized, phase 3)	NCT02477826	combo	> 10	based on NCT02659059	NA	PFS	1004
Rizvi, 2018 [42]	MSK-IMPACT (v1, v2, v3)	NSCLC	trial (randomized, phase 1)	NCT01295827	PD-(L)1	Q2 (7.4)	distribution	AUC = 0.601 (DCB)	DCB, PFS	240
Ready, 2019 [28]	FM1 CDx	NSCLC	trial (non-randomized, phase 2)	NCT02659059	combo	10	ROC	AUC (95% CI) = 0.73 (0.62–0.84); TPR (95% CI) = 0.78 (0.63–0.93); FPR (95% CI) = (0.62 (0.49–0.73)	ORR	98
Wang, 2019 [49] ^a^	NCC-GP150	NSCLC	observational (cohort)	NA	PD-(L)1	6 (tot mut)	best cutoff from in silico analysis on Rizvi 2015 WES	NA	PFS, ORR	50
Johnson, 2016 [12]	FM1 (v2, v3)	melanoma	observational (retrospective)	NA	PD-(L)1	< 3.3, 3.3–23.1, > 23.1	ROC	NA	PFS, OS, ORR	65
Chalmers, 2017 [22]	FM1 (v1, v2, v3, v4), FM1 Heme	various locally advanced or metastatic solid tumors	observational (retrospective)	NA	NA	> 20	NA	NA	NA	102, 292
Goodman, 2017 [18]	FM1 (v1, v2, v3)	various locally advanced or metastatic solid tumors	observational (cohort, retrospective)	NCT02478931	PD-(L)1, CTLA-4, high-dose IL2 or combo	< 6, 6–19, > 19	Foundation Medicine official reports	NA	PFS, OS, ORR	151
Khagi, 2017 [44] ^a^	Guardant360	various solid tumors	observational (cohort, retrospective)	NCT02478931	PD-(L)1, CTLA-4, combo or other	mean (> 3 VUS)	distribution	NA	PFS, OS, ORR	69
Zehir, 2017 [73]	MSK-IMPACT (v1, v2)	various primary and metastatic solid tumors	observational (cohort, prospective)	NCT01775072	NA	> 13.8	distribution (median TMB + 2 × IQR_TMB)	NA	NA	10, 945
Samstein 2019 [43]	MSK-IMPACT (v3)	bladder	observational (cohort, prospective)	NCT01775072	PD-(L)1, CTLA-4 or combo	17.6	distribution (top 20%)	NA	OS, PFS, DCB	214
		breast				5.9				45
		breast ER+				6.8				24
		breast ER-				4.4				21
		unknown primary				14.2				90
		colorectal				52.2				110
		esophagogastric				8.8				126
		glioma				5.9				117
		head and neck				10.3				138
		melanoma				30.7				321
		NSCLC				13.8				350
		renal cell carcinoma				5.9				151

*ORR* Objective Response Rates, *DCB* Durable Clinical Benefit, *OS* Overall Survival, *PFS* Progression-Free Survival, *FM1* Foundation Medicine’s FoundationOne (*v1*: 185 genes, *v2*: 236 genes, *v3*: 315 genes, *v4*: 405 genes, *Heme*: 405 genes, *CDx*: 324 genes); ±: version not specified; *MSK-IMPACT v1* 341 genes, *v2*: 410 genes, *v3* 468 genes, *NSCLC* non-small cell lung cancer, *ER* Estrogen Receptor, *VUS* variants of unknown significance, *PD-(L)1* anti-PD-1 or anti-PD-L1, *CTLA-4* anti-CTLA-4, *combo* combined anti-PD-1/PD-L1 + anti-CTLA-4, *Q1-Q4* quartiles, ^a^: TMB quantification from blood

Each study is described reporting gene panel, cancer type, study design, study ID (on ClinicalTrials.gov), immune checkpoint inhibitor treatment (ICI), proposed TMB cutoff, method for TMB cutoff determination, outcome analyzed to evaluate TMB clinical utility. AUC, TPR (True Positive Rate) and FPR (False Positive Rate) are provided, when available, as a measure of TMB predictive value for immunotherapy responder classification

The main challenge for accurate panel-based TMB quantification is the ability to extrapolate the global mutational burden from the narrow sequencing space targeted by a gene panel. In silico analyses were performed to test the concordance between panel-based and WES-based TMB, which is considered the reference for TMB quantification. Publicly available WES datasets were downsampled to the subset of genes targeted in the panel under consideration and TMB values from such simulated gene panels were compared with TMB values from the original WES (Additional file 7: Figure S1), finding high correlation between the two (Additional file 2: Table S2, Additional file 8: Figure S2). Most of these in silico analyses were performed using publicly available WES datasets from TCGA, with the exception of the Oncomine Tumor Mutation Load Assay or NovoPM and CANCERPLEX gene panels, for which WES datasets from COSMIC or from other sources were used. Regardless, similar correlation values were reported for the different gene panels tested (Additional file 2: Table S2, Additional file 8: Figure S2). For some of these gene panels (FoundationOne, Trusight170, Oncomine Tumor Mutation Load Assay, Oncomine Comprehensive Assay V3 and MSK-IMPACT gene panels), an empirical approach was also used to test the concordance between panel-based and WES-based TMB quantification, based on matched sequencing by gene panel and WES of the same tumor sample and comparison of matched TMB values (Additional file 3: Table S3, Additional file 9: Figure S3). Accuracy of panel-based TMB quantification is influenced by statistical sampling effects and small panels provide less precise TMB estimates [22, 34–36]. It was demonstrated that TMB values from the FoundationOne gene panel, which targets 1.1 Mb of genomic space, are similar to those from WES, whereas accuracy drops importantly when sequencing less than 0.5 Mb [22]. Another study simulated sequencing of theoretical gene panels of different sizes and identified 1.5 to 3 Mb as the best suited targeted genomic size to confidently estimate TMB [35]. Moreover, the deviation between WES- and panel-based TMB appears more relevant for samples with low to moderate underlying TMB levels, compared to samples with high TMB [22, 35, 36]. Another retrospective study on a commercial panel of 248 genes likewise cautions against small gene panels which would lead to TMB overestimation [37].

**Fig. 2:**
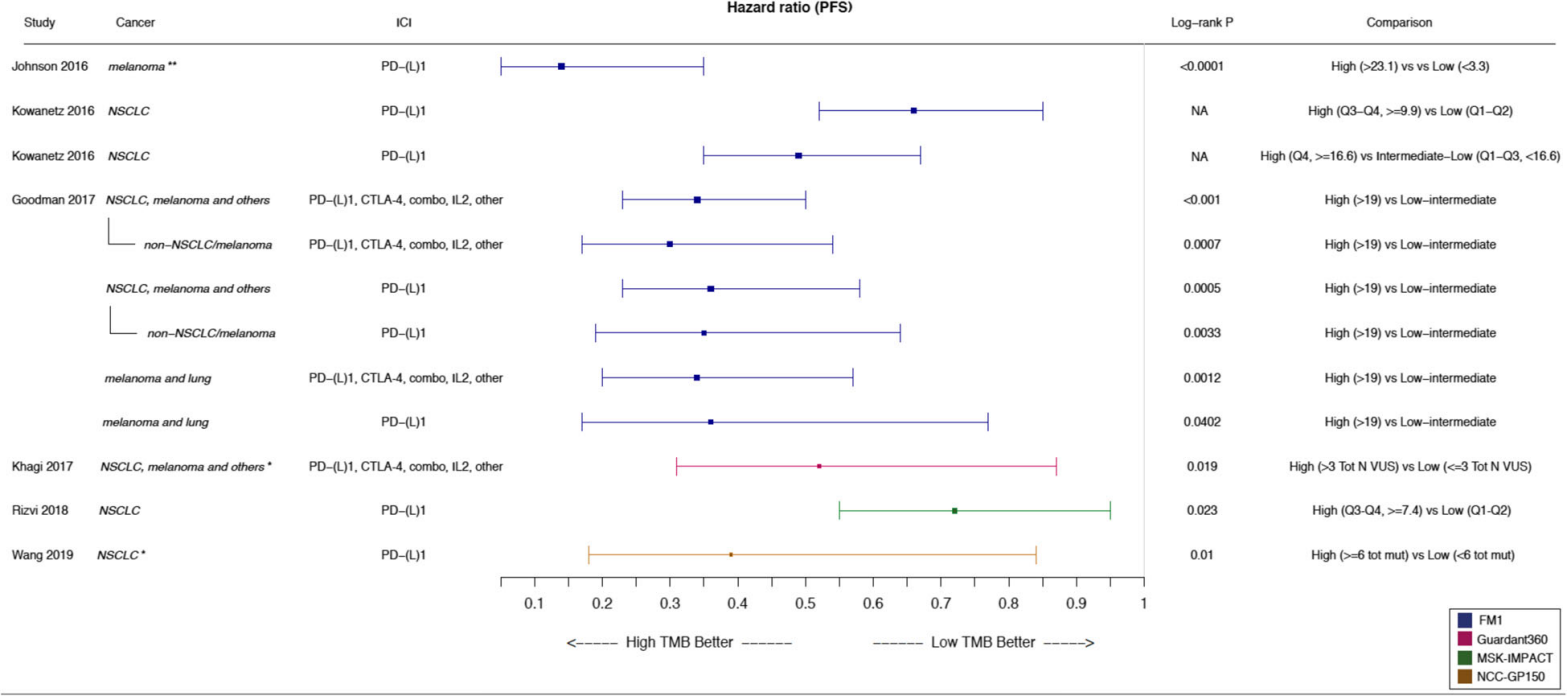
TMB association with progression-free survival. Forest plot of hazard ratios (HR) comparing progression-free survival (PFS) between patients with high or low TMB, as indicated in the “Comparison” column. If not specified otherwise, TMB is reported as number of mutations per Mb. All patients were treated with immune checkpoint inhibitors (*ICI*). Bars represent the 95% confidence intervals. Size of the box is proportional to precision. Reference to the study and the analyzed cancer type are also reported together with the log-rank *p*-value. *Q1-Q4*: quartiles; *VUS*: variants of unknown significance. *: TMB quantified from blood; **: Cox proportional hazards model adjusted for age, gender, disease stage and prior therapy by ipilimumab

Besides the accuracy of panel-based TMB quantification, it is critical to assess its capability to discriminate between immunotherapy responders and non-responders, as previously observed for WES-based TMB. Several exploratory analyses demonstrated that panel-based TMB, as simulated in silico by downsampling a WES dataset to only include genes targeted by the FoundationOne gene panel, associates with immunotherapy response [8, 26] or with signatures of immune checkpoint activation [38]. Comparable results were observed in similar in silico analyses for other gene panels, such as the Trusight170 [39, 40] or MSK-IMPACT [26] (Additional file 4: Table S4). Notably, direct measurement of TMB from the Oncomine Tumor Load Assay shows that this panel-based TMB value allows to classify colorectal cancer cases based on their MSI status [39, 41]. Since in this cancer type MSI positively correlates with immunotherapy response, this is a further, yet indirect evidence, of the capability to predict immunotherapy response, using a panel-based TMB estimate. Most importantly, a few clinical studies demonstrated that TMB directly estimated using gene panels is higher in those patients who benefit more from immune checkpoint blockade treatment, thus providing “real-life” evidence for its potential clinical predictive value (Fig. 2, Additional file 5: Table S5). A direct association with immunotherapy response was shown for the MSK-IMPACT [42, 43] and the Guardant360 gene panels [44] but most of the reported studies utilized the FoundationOne gene panel (Fig. 2, Additional file 5: Table S5). In particular, in the CheckMate 227 trial, NSCLC patients with high TMB (> 10 mutations per Mb, measured by FoundationOne) presented increased progression-free survival after combined anti-CTLA-4 plus anti-PD-1/PD-L1 therapy [45]. Interestingly, TMB was predictive of anti-PD-L1 monotherapy response in NSCLC (POPLAR trial, [27]) and metastatic urothelial carcinoma patients [5, 15, 16], independently from the PD-L1 expression status. Analysis of archival tumor samples from melanoma patients treated by anti-PD-1/PD-L1 monotherapy also showed superior response rates, progression-free survival and overall survival in high TMB cases [12]. Moreover, a retrospective study on 151 patients across diverse tumor types showed that cancer patients with higher TMB, benefit more from anti-PD-1/PD-L1, anti-CTLA-4 or high dose IL2 monotherapy [18]. The same was not observed for combined anti-PD-1/PD-L1 plus anti-CTLA-4 therapy but the available number of samples may be too small to draw conclusions [18].

## Need for standardization of TMB quantification and reporting

Despite the increasing number of studies showing the potential clinical relevance of panel-based TMB as a predictive biomarker for immunotherapy response, its use in the clinical setting is currently limited by the absence of standard methods of quantification and the lack of a robust and universal cutoff to identify immunotherapy responders.

**Fig. 3:**
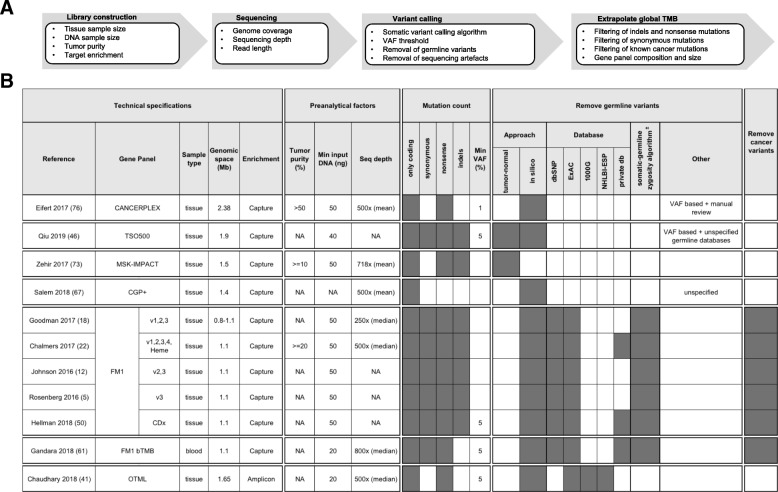
Differences in the workflow for panel-based TMB quantification. **a**. Overview of the factors influencing panel-based TMB quantification. Several variables in library construction, sequencing and in the pipeline to call mutations influence panel-based TMB quantification. Furthermore, panel-based TMB quantification is influenced by differences in the bioinformatic method to extrapolate global TMB from mutations identified in the narrow genomic region targeted by the gene panel. **b**. Differences across various studies in panel-based TMB quantification: gene panel technical specifications, preanalytical factors and the bioinformatics workflow used to extrapolate from the genomic space targeted by gene panels global TMB are described. *FM1*: Foundation Medicine’s FoundationOne panel (v1: 185 genes, v2: 236 genes, v3: 315 genes, v4: 405 genes); *NA:* not available; *±*: algorithm developed by Sun et al. for in silico removal of germline variants [74]

Panel-based TMB quantification is influenced by various experimental factors affecting library construction and sequencing, by the pipeline used to call mutations and by the capability to extrapolate TMB values from the restricted genomic space sampled by gene panels to the whole genome (Fig. 3a). Experimental factors (e.g. tumor purity or sequencing depth) and the variant calling pipeline (e.g. the variant calling algorithm and the method to remove germline variants) can significantly affect the number of called somatic mutations and have a similar impact on both panel-based and WES-based TMB quantification. Indeed, the adoption of a well-documented standard pipeline was already claimed for WES analyses as an urgent need to allow data interoperability between different platforms [46]. The same applies to panel sequencing for TMB quantification. In this context, an important factor investigated for its influence on the number of called somatic variants is the method chosen to identify and remove germline variants. Indeed, since only somatic mutations can potentially produce tumor neoantigens recognized as non-self by the immune system, it is important to remove germline variants in TMB quantification. It was observed that the use of an in silico method for somatic variant calling instead of matched tumor-normal samples, leads to increased false positive somatic variants, which has an important influence on the accuracy of TMB quantification, especially for small gene panels [34]. To avoid this, it was proposed to perform TMB quantification using only high-confidence regions [47] (e.g. regions of the genome, devoid of potential systematic biases or structural variants, where mutations can be confidently called), as defined by Zook et al. [48]. It was also observed that increased somatic false positives are generated by the in silico germline filtering method for patients with non-caucasian ancestry compared to caucasian patients, as the former are less represented in public databases used for germline variant filtering [34]. The use of ExAC, the largest and more representative public germline WES database, in addition to dbSNP and 1000 Genomes, is recommended to reduce this difference [34].

In addition to these factors, which similarly influence WES- and panel-based analyses, panel-based TMB quantification also requires to extrapolate the number of somatic coding mutations observed in the targeted genomic space to the number that would be observed across the whole genome. Extrapolation methods may differ for various choices in variant filtering, such as removal of known cancer mutations or synonymous mutations (Fig. 3b). Standard gene panels are commonly enriched in known cancer genes, which are more likely to be mutated in a tumor and expectedly enriched in mutations. Therefore, it was proposed to remove known cancer variants of targeted genes when performing TMB quantification, to avoid overestimation of TMB when extrapolating it across the whole genome [12, 22]. Buchhalter et al., showed that removal of cancer mutational hotspots slightly decreases the number of high TMB tumors identified but does not change the general picture [35]. However, the importance of this filtering, routinely performed only for Foundation Medicine panels, depends on panel size and composition: some gene panels may be larger and less enriched in cancer genes by including, for example, pharmacogenomic variants. As for synonymous mutations, it is claimed that, although not biologically meaningful, their inclusion may reduce sampling noise and improve the approximation of TMB across the whole genome. Indeed, several works compared TMB quantification with or without synonymous variants and observed that, when including synonymous variants, panel-based TMB shows increased correlation with WES-based TMB values [35, 49] and stronger association with clinical response [9]. Starting from the above observations, we can infer that some recommendations to build a standardized and robust analysis pipeline for TMB quantification are starting to emerge at least for the following points: i. germline variants can be most efficiently identified and removed by matched normal sequencing and, if this is not possible, the largest available germline variant databases should be used for in silico filtering, especially for non-caucasian ancestries; ii. TMB extrapolation to the whole genome is accurately performed by counting all somatic mutations, including synonymous mutations, to enlarge the sampling space and better approximate global TMB across the whole genome.

As already mentioned, panel-based TMB quantification is affected by the genomic size targeted by the panel and by its gene composition. Notably, gene panels tested up to now widely differ for number of targeted genes (from 73 to 710) and size (from 0.39 to 2.8 Mb of targeted genomic space) (Additional file 1: Table S1). These considerations raise the question of how to convert TMB estimates between different gene panels to allow cross-platform comparability. Indeed, although the majority of studies correlating TMB to immunotherapy response are currently based on FoundationOne, several other types of gene panels exist and the offer is steadily increasing (Additional file 1: Table S1). Moreover, we still need to standardize the conversion of the reference WES-based TMB values to panel-based TMB, since the lower sequencing coverage and higher sequencing depth of gene panels, as compared to WES, may lead to decreased accuracy of TMB values and increased sensitivity in variant calling. For cross-panels or panel-to-WES TMB conversion, an in silico approach was proposed, where TMB distributions derived from two different technologies were interpolated and aligned and TMB cutoffs were mapped across distributions [38]. However, a consensus on a standard method to convert TMB values is still missing.

Not surprisingly, in this heterogeneous landscape a robust TMB cutoff to discriminate between immunotherapy responders and non-responders is still to be defined. Moreover, the adopted cutoffs sometimes differ across different studies on the same gene panel (Table 1). Up to now, the TMB cutoff of 10 mutations per Mb, measured by the FoundationOne gene panel and found to best discriminate between responders and non-responders to immunotherapy in NSCLC patients, is the only one which has been validated in a separate further study [28, 50, 51]; this cutoff was also observed, but not yet validated, in melanoma [38] and in metastatic urothelial carcinoma [15] (Table 1). Interestingly, these cancer types present a TMB distribution similar to that of NSCLC [52]. Indeed, due to the diversity of TMB distribution across different cancer types, the adoption of cancer-specific TMB cutoffs was proposed [35, 43]. TMB cutoff was initially most commonly established using distribution-based stratification, which can be heavily influenced by outliers, while it is now often identified based on statistically sound methods, such as ROC curves. In the future, the use of ROC curves as a common method of TMB cutoff determination will greatly help to converge to a robust TMB cutoff and will facilitate comparison across different platforms.

The heterogeneity in experimental and analytical protocols, in the extrapolation of panel-based TMB values and in gene panel technical specifications is currently limiting the potential use of TMB in a clinical setting (Fig. 3). For this reason, a common standard for TMB quantification and a consensus on a clinically useful TMB cutoff are urgently needed. Some efforts in this sense are ongoing by the Quality Assurance Initiative Pathology (QuIP) in Germany (https://quip.eu) and by the European Society of Pathology. Moreover, in the US, governmental organizations, health-sector industries, several NGS gene panel manufacturers and academic institutions set up a TMB Harmonization Working Group (https://www.focr.org/tmb) and planned a 3-phase project for TMB harmonization. In the first phase, they performed in silico analyses of publicly available TCGA data to identify sources of variability in TMB quantification between WES and gene panels. Recently concluded, this work established that panel-based TMB is comparable between different gene panels for TMB values ranging 0 to 40 mutations per Mb, that it strongly correlates with WES-based TMB and it is possibly influenced by the type of cancer under investigation. It also found that the observed variance across gene panels stems from their different gene composition and technical specifications, as well as from the bioinformatic pipeline adopted [53]. The second step of the project empirically validates TMB estimates from different gene panels by mapping them to WES-based TMB values, used as a gold standard, whereas the last step will define best practices for TMB use as immunotherapy biomarker in clinical routine. Following preliminary results on the influence of the bioinformatic workflow and of gene panel size and composition on TMB quantification, the working group recommends the use of gene panels larger than 1 Mb and the standardization of the bioinformatic algorithms, in addition to standardization of sample processing. Moreover, it suggests the inclusion of actionable genes, genes associated with mutagenesis and negative predictors of response in these gene panels and the alignment of panel-based TMB values to WES-based ones to allow interoperability across different assays [54].

## TMB quantification beyond tissue biopsies and current gene panels

Most studies on TMB as a predictive biomarker for immunotherapy response were performed on bioptical or surgical specimens from solid tumors. Since obtaining tissue biopsies may be challenging and invasive for patients, it would be critical for the clinical routine to assess TMB using cell-free DNA (cfDNA) from blood, which includes circulating tumor DNA (ctDNA), as a surrogate specimen to biopsy. High throughput molecular profiling of ctDNA remains technically challenging but increasing efforts are being made in this direction. A few studies previously investigated the feasibility of WES on ctDNA and highlighted some inherent limitations, such as the low amount of available ctDNA, which reduces sensitivity, or ctDNA being more associated with metastases rather than with primary tumors [55–59]. In one of the largest studies attempting optimization of WES-based TMB quantification from liquid biopsy, WES was performed in parallel on DNA from tissue biopsies and on cfDNA from liquid biopsies of 32 metastatic patients and comparable sequencing depth and coverage were obtained [60]. Performance of variant detection was dependent on the fraction of tumor DNA within the analyzed cfDNA, as previously described. In those samples positive for the presence of ctDNA, variant detection sensitivity of cfDNA-WES compared to tDNA-WES was 94%, regardless of the tumor type (2 cholangiosarcoma and 19 lung, 5 head and neck, 2 prostate, 2 colorectal, 1 breast and 1 bladder cancer were analyzed). Most importantly, in ctDNA positive samples, TMB values from WES on liquid biopsies were robust and consistent with those from WES on tissue biopsies, which demonstrates for the first time the feasibility of TMB quantification from liquid biopsies, using WES.

More recently, it was demonstrated that targeted enrichment sequencing by gene panels is another valid approach for TMB quantification from liquid biopsies. In particular, Gandara et al. developed, tested and analytically validated a novel gene panel for TMB quantification from blood [61]. The panel is based on hybridization-capture enrichment and targets 394 genes, corresponding to 1.1 Mb of genomic space (Fig. 3). Its clinical utility was evaluated via a retrospective study on 259 NSCLC samples from patients treated with immunotherapy or chemotherapy in the OAK and POPLAR clinical trials. Blood-derived TMB (bTMB) calculated using this novel gene panel correlated well with tissue-derived TMB (tTMB) measured by FoundationOne. Moreover, measured TMB was found to be significantly associated with response to anti-PD-L1 immunotherapy in the POPLAR trial and this was further confirmed on patient samples from the OAK trial. A prospective validation is also currently ongoing in the BFAST trial (NCT03178552) on advanced and metastatic NSCLC patients. Interestingly, it was observed that the capability of TMB, as measured by this panel, to predict anti-PD-1/PD-L1 immunotherapy response is independent from PD-L1 expression levels [61]. One of the main pitfalls of the panel, though, is its limit of detection, defined as a minimum of 1% tumor content in at least 20 ng of cell-free DNA input, and its dependency on the overall tumor burden, which influences the likelihood of detecting ctDNA. The exclusive use of single nucleotide variants (SNVs) for TMB quantification represents another limitation, although future versions of the algorithm are planned to be released, which will also use indels. The commercial Guardant360 and GuardantOMNI gene panels were also designed for blood-based TMB quantification [62]. Their limit of detection was defined as a minimum of 0.3% tumor content in at least 5 ng of cell-free DNA input. They were validated in silico by subsetting TCGA WES datasets to only include genes targeted by the panels. Panel accuracy in TMB quantification was then evaluated by correlation of TMB values obtained from the simulated gene panels with those from WES. Their predictive value was similarly evaluated in silico on 30 lung cancer samples with matched information on immunotherapy response. The performance showed by TMB from the simulated gene panel in responder identification was comparable to that of WES-based TMB (Additional file 4: Table S4). The Guardant360 panel was further tested in a small retrospective study on 69 patients with various tumor types [44]. No comparison with tissue-based TMB has been reported yet, but a significant correlation between high blood-derived TMB measured by Guardant360 and immunotherapy response was observed [44]. Finally, a further gene panel for bTMB quantification was recently developed in China. Consistency between panel-based bTMB values and WES-based tTMB values, tested in silico and empirically by matched blood and tissue samples, was comparable to that of the panels described above. Similar results were also found for its predictive value, based on in silico analyses. Interestingly, the authors also raised the issue of the different frequency of oncogenic driver mutations, such as EGFR or KRAS, between Asian and white population. For this reason, they compare TCGA WES-based TMB with panel-based TMB with or without inclusion of EGFR and/or KRAS mutations. Although similar results are yielded, the raised issue is an important point to be further investigated in panel-based TMB quantification [49]. TMB quantification from liquid biopsies suffers from ctDNA detection limits, which also depend on tumor size and number of cancer cells, but these results encourage to further explore and more extensively validate this approach.

Besides new technologies to estimate TMB from liquid biopsies, another significant step towards routine use of TMB in clinical practice is TMB quantification from an even smaller set of genes than in targeted enrichment gene panels. Although panel size is known to affect accuracy of TMB quantification, the use of a highly customized set of genes may represent a valid and even less expensive approach. In this view, Lyu et al., proposed a computational framework to assess the best and smallest subset of genes necessary to estimate TMB as a biomarker for lung adenocarcinoma [63]. They were able to identify a model of only 24 genes which predicted in silico immunotherapy response with 93% specificity and 85% sensitivity and they suggested that other small custom sequencing gene panels may be designed in a cancer-specific way to assess TMB with further reduced costs.

## Future perspectives and recommendations

TMB is one of the most rapidly developing biomarkers for immunotherapy response, with about 37 ongoing clinical trials currently registered in ClinicalTrials.gov that use TMB as stratification biomarker [64]. Several gene panels were recently optimized to estimate TMB at reduced sequencing costs, and emerging evidence supports the feasibility of TMB quantification from liquid biopsies. However, harmonization in TMB quantification and reporting remains the main challenge for the near future: standard procedures are required to allow interoperability between different gene panels, compare results across studies and define a universal cutoff to confidently identify patients most likely to benefit from immunotherapy.

Even an accurate TMB value is an imperfect predictor of immunotherapy response and further studies are needed to enhance its value as clinically useful immunotherapy biomarker. TMB is used as an approximation of neoantigen burden, upon the assumption that the higher the mutational burden, the higher the probability for immunogenic peptides to be generated, which leads to stronger immune response upon inhibition of immune checkpoints. Interestingly, neoantigen clonality, in addition to the overall amount of neoantigens, influences immunotherapy response in NSCLC patients [65]. In particular, tumors enriched in clonal neoantigens (e.g. present in all tumor cells) are more sensitive to immune checkpoint inhibitors than tumors enriched in subclonal neoantigens (e.g. present only in a subset of tumor cells), in advanced NSCLC and melanoma patients [65]. Indeed, clonality of produced neoantigens seems to be associated with a more effective immune surveillance. On the other hand, enrichment in subclonal neoantigens may activate T cells against only a subset of tumor cells, leading to less effective tumor control. Based on these observations, it would be interesting to investigate if information on mutation clonality (e.g. variant allele frequency) improves the predictive power of TMB. Evaluation of mutation clonality from gene panels is not trivial though: the reduced genomic space targeted by gene panels may not be representative of the overall clonal architecture and the mutations sampled herein may not be those generating neoantigens. Interestingly, McGranahan et al. observed a relationship between subclonal mutations and mutational signatures associated with alkylating agents and, in NSCLC, between clonal mutations and mutational signatures associated with smoking [65]. Mutational signatures associated with smoking were also found to be significantly associated with high tumor mutational burden and with response to immunotherapy [9]. Therefore, although the extraction of mutational signatures from gene panels may be hampered by the small number of sampled mutations, these observations suggest that they may prove helpful to infer neoantigen clonality and enhance TMB predictive value.

Integration of TMB with other potential immunotherapy biomarkers represents another promising way to refine prediction of immunotherapy responders. For example, TMB, defects in DNA mismatch-repair pathway and the MSI status all are measures of genomic instability that can provide indirect assessment of tumor antigenicity, whereas PD-L1 expression, immune cell infiltration and inflammatory signatures represent biomarkers of the T cell-inflamed tumor microenvironment. Therefore, their integration can refine prediction of immunotherapy outcome by combining information on tumor complexity and on the immune response. Indeed, emerging evidence suggests that, at least in NSCLC, TMB and PD-L1 expression are independent predictors and TMB may complement or even outperform PD-L1 expression [10, 26, 50, 66]. Moreover, it was observed that most tumors with high MSI also present elevated TMB, whereas the opposite does not hold true. The combination of TMB with MSI and PD-L1 expression in gastrointestinal tumors significantly improved the identification of immunotherapy responders [67]. In another study, it was observed that TMB is an independent predictor and only weakly correlates with T cell-inflamed gene expression profiles (GEP) or PD-L1 expression. Thus, TMB and T cell-inflamed GEP were jointly used to identify immunotherapy responders: patients with both high TMB and high T cell-inflamed GEP were those with the highest objective response rates on tumors from four KEYNOTE clinical trials across 22 cancer types. Similarly, in melanoma patients, a response score based on the combination of TMB, infiltration of CD8+ T cells and gene expression profiles for PD-L1, CD8 and a set of 394 immune genes demonstrated higher sensitivity and similar specificity than each biomarker alone [68]. To date, the FoundationOne and Guardant360 gene panels allow to measure both TMB and MSI but no other potential immunotherapy biomarker. Moreover, they do not provide the user any combinatorial model to integrate them. Although further validation in prospective clinical studies is required for all these potential biomarkers, several observations suggest that simultaneous profiling of both TMB and other immunotherapy biomarkers currently under investigation may represent the next step forward in the design of new gene panels for clinical use. The Friends and QuIP initiatives for TMB harmonization recommended to include as much relevant genetic and molecular information as possible in these panels, to avoid the need to re-biopsy the patient for further information. In line with this recommendation, we propose to also include in gene panels for TMB quantification other potential immunotherapy biomarkers but also negative predictors of immunotherapy response [69, 70] and variants predisposing to adverse reaction to immunotherapy [71, 72]. These and other recommendations which emerge from the studies reviewed here, including the one from the TMB Harmonization Working Group, are summed up in Additional file 6: Table S6.

## Additional files

Additional file 1: **Table S1.** Technical specifications of gene panels used or proposed for TMB quantification. For each gene panel, it is reported the type of cancer and sample for which it was designed, the enrichment method, the targeted sequencing size (Genomic space) and the number of targeted genes (# genes). (XLSX 6 kb)

Additional file 2: **Table S2.** In silico analysis of the correlation between panel-based and WES-based TMB. Correlation between panel-based and WES-based TMB, considered the gold standard value, is used to estimate the accuracy of panel-based TMB quantification. Panel-based TMB quantification was simulated in silico using a subset of WES which only contains genes targeted by the panel. (XLSX 11 kb)

Additional file 3: **Table S3.** Empirical analysis of the correlation between panel-based and WES-based TMB. Correlation between panel-based and WES-based TMB, considered the gold standard value, is used to estimate the accuracy of panel-based TMB quantification. Correlation analysis is performed on TMB values calculated for samples with matched panel and whole exome sequencing. (XLSX 6 kb)

Additional file 4: **Table S4.** In silico analysis of TMB association or predictive value for immunotherapy response. These analyses were performed on panel-based TMB values simulated in silico using a subset of WES which only contains genes targeted by the panel. The table reports measures of TMB association with immunotherapy response (odds ratios, hazard ratios and corresponding *p*-values), differences in TMB distribution between responders and non-responders (Mann-Whitney U and Fisher’s *p* values) and measures of TMB predictive value (AUC, specificity, sensitivity). (XLSX 9 kb)

Additional file 5: **Table S5.** Empirical analysis of TMB association or predictive value for immunotherapy response. These analyses were performed on panel-based TMB values, directly calculated by panel sequencing. The table reports measures of TMB association with immunotherapy response (odds ratios, hazard ratios and corresponding p-values), differences in TMB distribution between responders and non-responders (Mann-Whitney U, unpaired Student’s t and Fisher’s test p values) and measures of TMB predictive value (AUC, specificity, sensitivity). We also specify how patients were stratified (“Comparison”), the method used to determine TMB cutoff, the cohort considered for the analysis (if different cohorts were analyzed in the study), the type of immunotherapy, cancer type and number of patients. (XLSX 19 kb)

Additional file 6: **Table S6.** Proposed recommendations for consistent TMB quantification and reporting. We report recommendations formulated by the TMB Harmonization Working Group (https://www.focr.org/tmb) as well as indications emerging from the studies reviewed in this work. (XLSX 24 kb)

Additional file 7: **Figure S1.** Visual representation of the method used for in silico analyses on TMB quantification accuracy and on association or predictive value for immunotherapy response. In silico analyses are based on simulations of panel performance, wherein TMB is calculated using a subset of WES which only contains genes targeted by the panel. Accuracy of TMB quantification from the simulated gene panel is evaluated by comparison with WES-based TMB, used as gold reference, with correlation analysis. The clinical predictive value of TMB estimated from the simulated panel is evaluated based on its association with clinical values measuring immunotherapy response. (PDF 55 kb)

Additional file 8: **Figure S2.** In silico analysis of the correlation between panel-based and WES-based TMB. Correlation between panel-based and WES-based TMB, considered the gold standard value, is used to estimate the accuracy of panel-based TMB quantification. Panel-based TMB quantification was simulated in silico using a subset of WES which only contains genes targeted by the panel. The bubble plot shows on the x axis the correlation coefficients and on the y axis the gene panel and the cancer type. Bubble size represents the number of data points used in the analysis and the color corresponds to the reference study. (PDF 259 kb)

Additional file 9: **Figure S3.** Empirical analysis of the correlation between panel-based and WES-based TMB. Correlation between panel-based and WES-based TMB, considered the gold standard value, is used to estimate the accuracy of panel-based TMB quantification. Correlation analysis is performed on TMB values calculated for samples with matched panel and whole exome sequencing. The bubble plot shows on the x axis the correlation coefficients and on the y axis the gene panel and the cancer type. Bubble size represents the number of data points used in the analysis and the color corresponds to the reference study. (PDF 155 kb)

## Data Availability

Not applicable.

## Abbreviations

ACC: Adrenocortical carcinoma
AUC: Area under the curve
BLCA: Bladder urothelial carcinoma
BRCA: Breast invasive carcinoma
CESC: Cervical squamous cell carcinoma and endocervical adenocarcinoma
cfDNA: Circulating free DNA
CHOL: Cholangiosarcoma
COADREAD: Colon adenocarcinoma
CRC: Colorectal cancer
ctDNA: Circulating tumor DNA
DLBC: Lymphoid neoplasm diffuse large B-cell lymphoma
ESCA: Esophageal carcinoma
FDA: Food and Drug Administration
GBM: Glioblastoma
GEP: Gene expression profile
HLA: Human Leukocyte Antigen
HNSC: Head and neck squamous cell carcinoma
KICH: Kidney chromophobe
KIRC: Kidney renal clear cell carcinoma
KIRP: Kidney renal papillary cell carcinoma
LAML: Acute myeloid leukemia
LGG: Brain lower grade glioma
LIHC: Liver hepatocellular carcinoma
LUAD: Lung adenocarcinoma
LUSC: Lung squamous carcinoma
Mb: Megabase
mCRPC: Metastatic castration-resistant prostate cancer
MESO: Mesothelioma
MHC: Major histocompatibility complex
MMR: Mismatch repair
MSI: Microsatellite instability
NSCLC: Non-small cell lung cancer
ORR: Objective response rates
OS: Overall survival
OV: Ovarian serous cystadenocarcinoma
PAAD: Pancreatic adenocarcinoma
PCPG: Pheochromocytoma and paraganglioma
PFS: Progression free survival
PRAD: Prostate adenocarcinoma
ROC: Receiver operating characteristic
SARC: Sarcoma
SCLC: Small cell lung cancer
SKCM: Skin cutaneous melanoma
SNV: Single nucleotide variant
STAD: Stomach adenocarcinoma
TCGA: The cancer genome atlas
TCR: T cell receptor
TGCT: Testicular germ cell tumors
THYM: Thymoma
TMB: Tumor mutational burden
UCEC: Uterine corpus endometrial carcinoma
UCS: Uterine carcinosarcoma
UVM: Uveal melanoma
WES: Whole exome sequencing
