# Supplementary material for: Tumor mutational burden quantification from targeted gene panels: major advancements and challenges
Source: J Immunother Cancer. 2019 Jul 15;7:183. doi: 10.1186/s40425-019-0647-4 (PMC6631597; doi:10.1186/s40425-019-0647-4)
Supplement: Supplementary file 7 — Figure S1. Visual representation of the method used for in silico analyses on TMB quantification accuracy and on association or predictive value for immunotherapy response. In silico analyses are based on simulations of panel performance, wherein TMB is calculated using a subset of WES which only contains genes targeted by the panel. Accuracy of TMB quantification from the simulated gene panel is evaluated by comparison with WES-based TMB, used as gold reference, with correlation analysis. The clinical predictive value of TMB estimated from the simulated panel is evaluated based on its association with clinical values measuring immunotherapy response. (PDF 55 kb) [file 40425_2019_647_MOESM7_ESM.pdf]

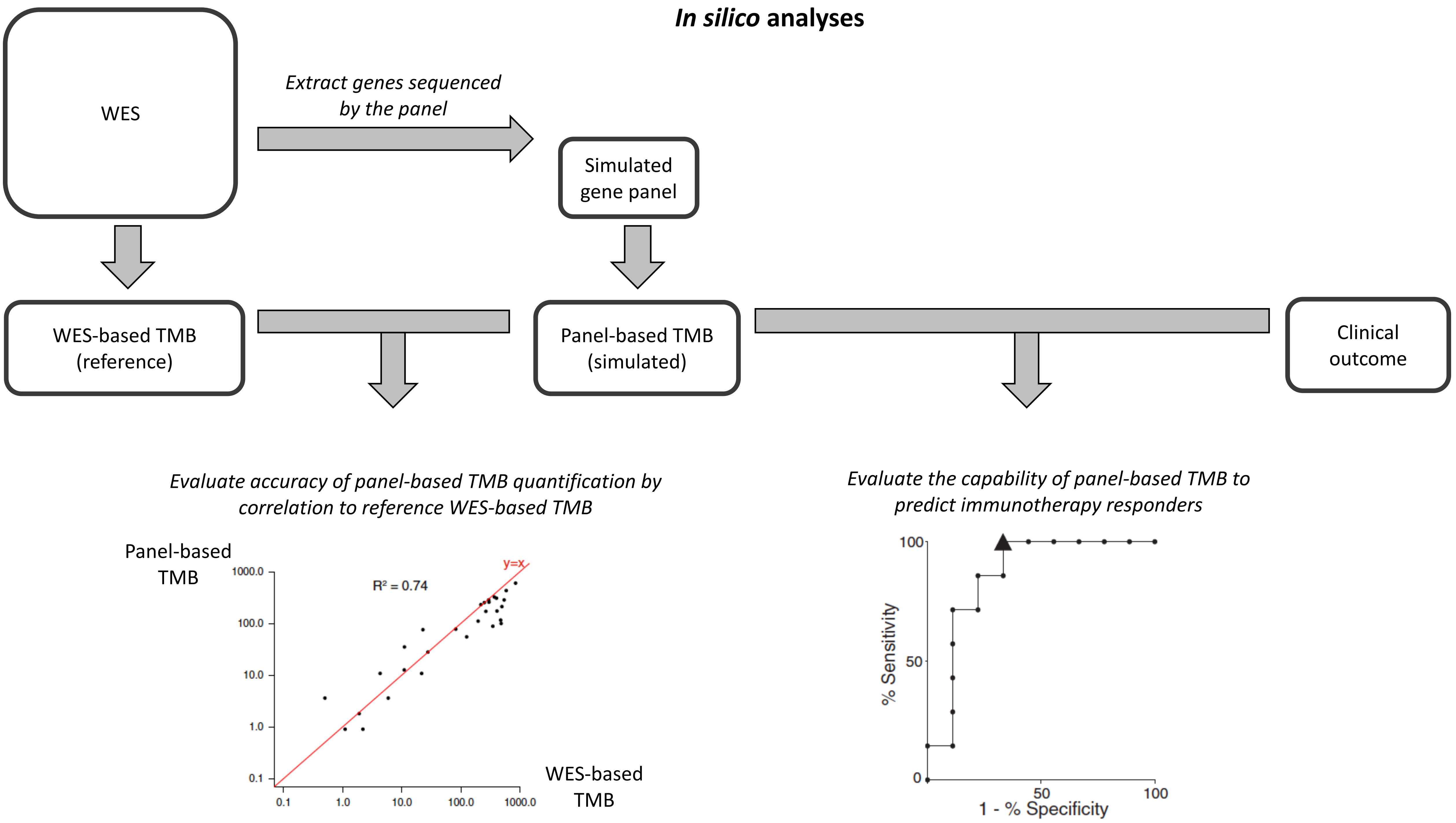

In silico analyses
WES
Extract genes sequenced by the panel
Simulated gene panel
WES-based TMB (reference)
Panel-based TMB (simulated)
Clinical outcome
Evaluate accuracy of panel-based TMB quantification by correlation to reference WES-based TMB
Evaluate the capability of panel-based TMB to predict immunotherapy responders
Panel-based TMB
WES-based TMB
R² = 0.74
y=x
1000.0
100.0
10.0
1.0
0.1
% Sensitivity
1 - % Specificity
100
50
0
