# Supplementary figures and images for: Tumor mutational burden quantification from targeted gene panels: major advancements and challenges
Source: J Immunother Cancer. 2019 Jul 15;7:183. doi: 10.1186/s40425-019-0647-4 (PMC6631597; doi:10.1186/s40425-019-0647-4)

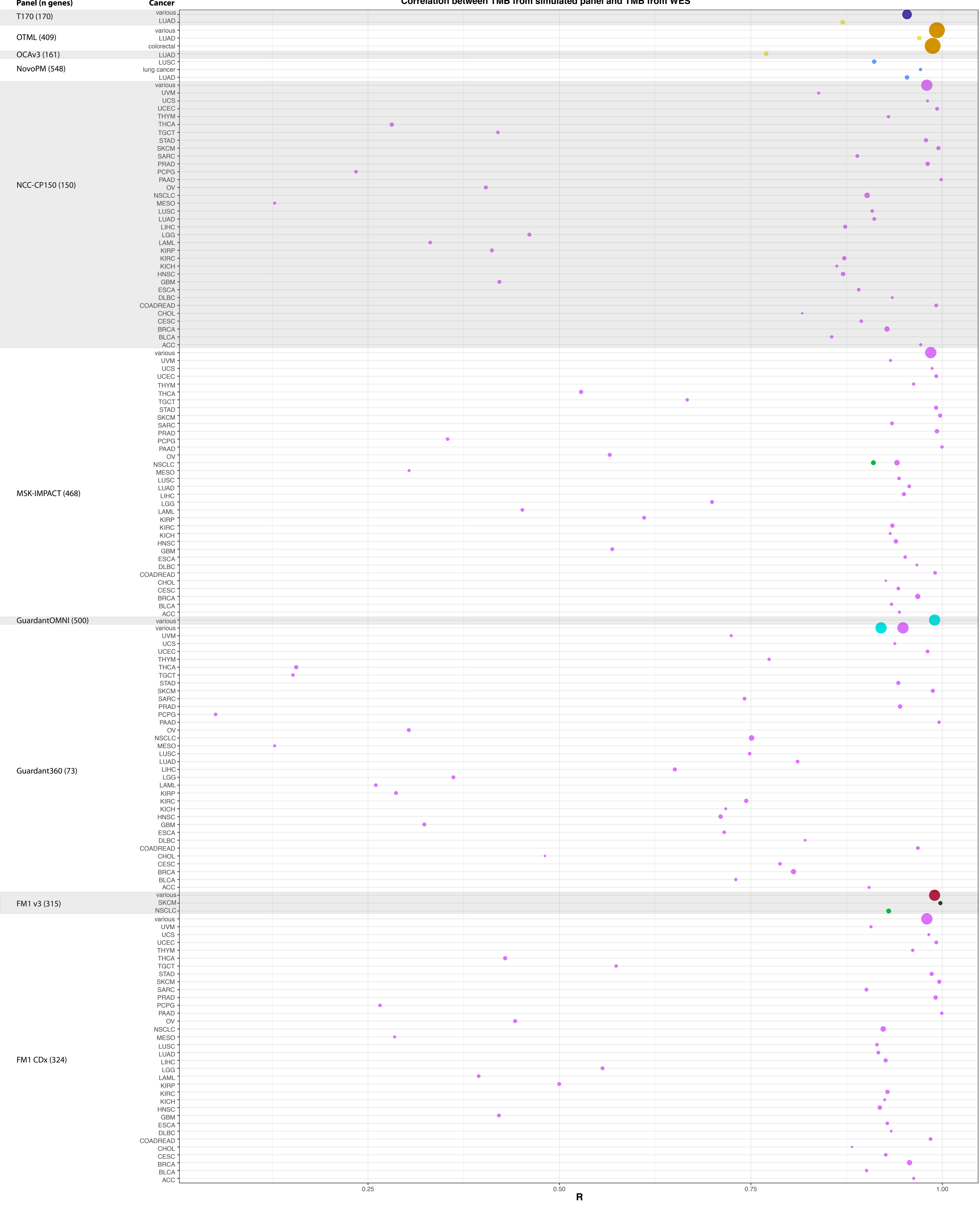

Supplement: Supplementary file 8 — Figure S2. In silico analysis of the correlation between panel-based and WES-based TMB. Correlation between panel-based and WES-based TMB, considered the gold standard value, is used to estimate the accuracy of panel-based TMB quantification. Panel-based TMB quantification was simulated in silico using a subset of WES which only contains genes targeted by the panel. The bubble plot shows on the x axis the correlation coefficients and on the y axis the gene panel and the cancer type. Bubble size represents the number of data points used in the analysis and the color corresponds to the reference study. (PDF 259 kb) [file 40425_2019_647_MOESM8_ESM.pdf]

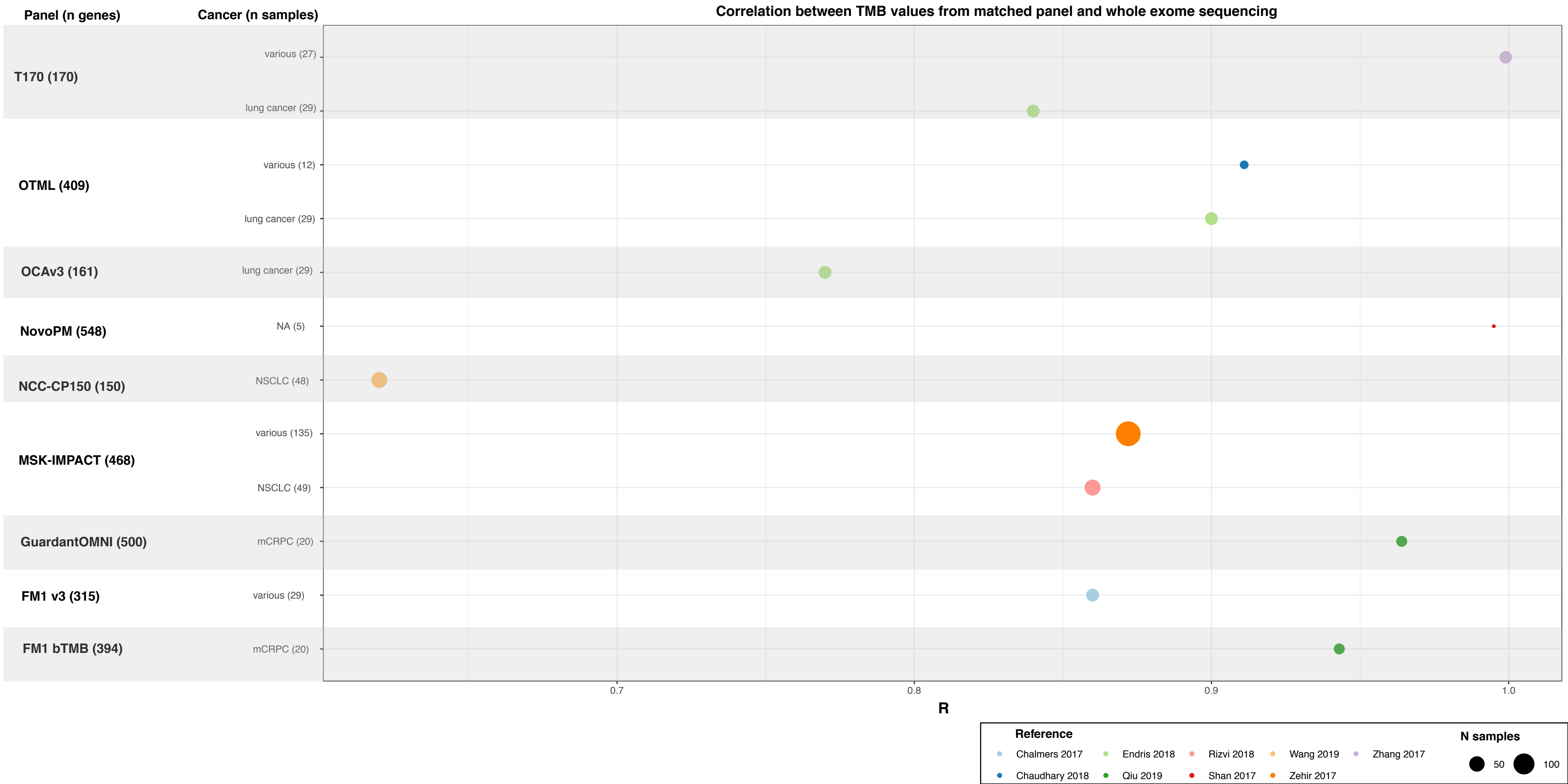

Supplement: Supplementary file 9 — Figure S3. Empirical analysis of the correlation between panel-based and WES-based TMB. Correlation between panel-based and WES-based TMB, considered the gold standard value, is used to estimate the accuracy of panel-based TMB quantification. Correlation analysis is performed on TMB values calculated for samples with matched panel and whole exome sequencing. The bubble plot shows on the x axis the correlation coefficients and on the y axis the gene panel and the cancer type. Bubble size represents the number of data points used in the analysis and the color corresponds to the reference study. (PDF 155 kb) [file 40425_2019_647_MOESM9_ESM.pdf]
